# Age Differences in Leadership Positions Across Cultures

**DOI:** 10.3389/fpsyg.2021.703831

**Published:** 2021-09-17

**Authors:** Thomas Vaughan-Johnston, Faizan Imtiaz, Albert Lee, Li-Jun Ji

**Affiliations:** ^1^Department of Psychology, Queen’s University, Kingston, ON, Canada; ^2^Department of Psychology, Durham University, Durham, United Kingdom; ^3^Department of Psychology, Towson University, Towson, MD, United States; ^4^School of Social Sciences, Nanyang Technological University, Singapore, Singapore

**Keywords:** leadership, aging, culture, cultural tightness, business, politics

## Abstract

In most countries around the world, the population is rapidly aging. A by-product of these demographic shifts is that older adults will likely occupy more positions of power and influence in our societies than ever before. Further, cultural differences might shape how these transitions unfold around the globe. Across two studies, we investigated whether business and political leaders differed in age across various cultures. Study 1 (*N* = 1,034) showed that business leaders were significantly older in Eastern (e.g., China, India, and Japan) cultures than Western (e.g., United States, Sweden, and United Kingdom) cultures, even while controlling for population structure (e.g., percentage of elderly in the society), gross domestic product (GDP), and wealth distribution across the population (GINI). Study 2 (*N* = 1,268) conceptually replicated these findings by showing that political leaders were once again older in Eastern vs. Western cultures. Furthermore, cultural tightness mediated the relationship between culture and older leadership. These findings highlight how cultural differences impact not only our preferences, but also important outcomes in consequential domains such as business and politics. Potential explanations for why cultural tightness may be related to differences in leader age across cultures are discussed. To build on these findings, future research should assess the potential causal mechanisms underlying the cultural effect on leader age, and explore the various practical implications of this effect.

## Introduction

The population is aging rapidly in many countries around the world, and these demographic shifts have major implications on our economies (Bloom et al., 2011), health care systems (Hashimoto and Tabata, 2010), retirement and pension plans (Gruber and Wise, 1999; Burtless, 2013), as well as business practices (Phillips and Siu, 2012). Accordingly, older people are now playing a more significant role in society compared to any other time period in modern history. As such, are older people more likely to take on leadership positions in some cultures than in others? If so, what might be the underlying mechanism? The present paper explores these questions, focusing on the effect of culture and aging on leadership positions.

### Aging and Leadership

One consequence of an aging world is that leadership positions are now held by older people more frequently than ever before. Psychological research has suggested that older leaders have some distinctive qualities compared to younger leaders. In a review of this literature, Truxillo and Burlacu (2015) asserted that the age of a leader or subordinate can significantly impact how they view and interact with one another. For instance, in one longitudinal field study examining this issue, Liden et al. (1996) reported that older leaders were able to produce superior objective performance (i.e., number of sales) among their subordinates compared to younger leaders. The researchers postulated that one reason for this effect may be that in some performance-based contexts, such as sales, older leaders are able to model superior performance for their subordinates, which, in turn, boosts their productivity. Along the same lines, Kearney (2008) found that age moderated the relationship between transformational leadership (comprised of charisma, inspirational motivation, intellectual stimulation, and individualized consideration; Barling et al., 2000) and group performance, such that transformational leadership was more likely to have a positive impact on group performance when the team leader was older than the team members. These findings suggest an association between age and leadership, with older leaders taken as a source of inspiration by their teams.

However, research has also shown that there may be important drawbacks to having older individuals leading team members who are significantly younger than them. For example, Malangwasira (2012) reported that age dissimilarity may lead to decreased job satisfaction through poor communication channels between older leaders and younger followers, as well as high role-ambiguity stemming from discrepancies in how older and younger individuals view the nature of work and their roles. Moreover, greater age discrepancy between a senior mentor and a young protégé is related to decreased agreement in their views of the partnership, likely due to meaningful differences in expectations and goals from the mentorship initiative (Fagenson-Eland et al., 2005).

In an attempt to reconcile these contradictory perspectives, Harrison et al. (2002) reported that although age differences between leaders and their followers do indeed produce some natural friction at the beginning of the relationship, this strain is reduced over time, and often eliminated as individuals come to know and understand one another in more meaningful ways (i.e., deep-level diversity), instead of making judgments based on superficial characteristics such as age (i.e., surface-level diversity).

Moreover, research has shown that age discrepancies between leaders and their followers do not necessarily produce tension, but rather this relationship is often dependent on additional factors such as how younger followers view aging more generally. More specifically, Zacher and Bal (2012) explored how views on aging in followers influenced the relationship between older leaders and their teams. Results indicated that differences in age between leaders and their followers led to negative ratings of the leader, but only when the followers harbored pre-existing negative beliefs about aging. In related work, researchers examined how leader generativity, or the degree to which a leader nurtures and guides the future generation, and how this plays a role in the links between leader age and team dynamics (Zacher et al., 2011). The findings indicated that the negative association between leader age and leader effectiveness was moderated by leader generativity, such that leaders who displayed care and concern for their younger team members were just as effective as younger leaders who were directing young teams.

Together, these findings reveal the value of a socio-cognitive look at age and leadership quality. Effective leadership seems to have less to do with how old the leader is compared to the team, and more to do with people’s beliefs about the elderly, along with older leaders’ approach to management. Assumptions about older people, however, are not the same around the world. Depending on culture, people may come to acquire distinct assumptions about the elderly, such as where the elderly are supposed to stand in society, who they represent, or what they can or cannot do.

### Cultural Views on Aging

To what extent can older individuals contribute to a society? Do they have the skills required to lead a large group of people on an important task? Or should they be on the receiving end of commands and orders? Answers to these questions have to do with the views or expectations people have about the elderly, which may vary across cultures. Cultural views on the elderly manifest themselves in concrete social contexts, shaping the way elderly people are perceived and treated by those around them. In the current work, the term “Eastern cultures” refers to countries in East and South Asia and the Middle East, and the term “Western cultures” refers to countries throughout Europe and North America.

In general, research has documented more positive views associated with the elderly in Eastern than Western cultures. For example, Sung (2001) argues that Confucian cultures (such as China, Japan, and Korea) cultivate positive ideals regarding elder respect. Specifically, Sung notes that Confucian teaching advocates for 14 forms of elder respect. Central to the present research are *consultative respect* (seeking elders’ wisdom on cultural issues), and *acquiescent respect* (obeying, not talking back). If “listen” is the word that captures the spirit of consultative respect, “do what I say” captures the spirit of acquiescent respect. In cultures where both forms of respect are in joint service, one may expect the old to have a stronger say on many things than the young, especially on decisions that are consequential to the group. This line of logic, one that stresses the normative and informational influence of the elderly on a society, is consistent with the core values of Confucian cultures, in which old age is venerated for the wisdom, experience, knowledge, and insight that it represents. By implication, older adults who are elevated with consultative and acquiescent respect in their culture should be acknowledged as the ones on the giving end of commands, not the ones taking them. The reverence that comes with old age, thanks to the assumptions of intellect behind it, is present in many South Asian cultures as well (Singh, 2005; Sung and Kim, 2009). These observations stand in sharp contrast to views of the elderly in the United States, many of which are relatively negative in terms of mental capacities (e.g., Rubin and Brown, 1975; Kite et al., 1991; Erber and Prager, 1999; Andreoletti et al., 2015). In fact, the negativity associated with older individuals in individualistic cultures is possibly growing worse over time (Ng et al., 2015).

Distinct views on the elderly between Eastern and Western cultures become clearer in cross-cultural research. Vauclair et al. (2017) compared Taiwanese and British citizens, and found cultural differences in social norms (i.e., more positive beliefs toward the elderly in Taiwan than in United Kingdom). Specifically, Taiwanese participants reported that most people in their culture view the elderly as more competent, with more admiration and envy (although with more contempt as well), compared to British participants. Taiwanese participants also associated old people with higher perceived social status and lower levels of perceived threat than did British participants, whereas British participants reported lower levels of direct and indirect prejudice and higher level of friendship with the elderly^1^.

Löckenhoff et al. (2009) recruited college students in 26 cultures to study their perceptions of age-related changes in physical, cognitive, and socioemotional functioning and societal views of aging within their cultures. There was little cultural variation in the perceptions of physical (e.g., physical attractiveness) and cognitive aging (e.g., performance of everyday tasks; learning new things), in that both domains were perceived to decrease with age regardless of the cultural backgrounds of participants. In contrast, more cultural differences emerged for socioemotional aspects of aging (i.e., received respect, family authority, and life satisfaction), and the strongest cultural differences emerged for societal views of aging. Specifically, participants in Eastern cultures reported more positive societal views of aging than those in Western cultures. In addition, the proportion of older adults (i.e., people aged 65 and above) in the population was associated with less favorable societal views of aging. Indeed, when controlling for the proportion of older adults in the population, East–West differences in societal views of aging became non-significant. This finding is important because it highlights how apparent cross-cultural differences in societal views of the elderly may be driven by differences in population structure (e.g., the percentage of elderly in a population).

Thus, most research seems to suggest that there are more positive views of the elderly in Eastern than Western countries, with a few findings indicating otherwise. Instead of examining people’s beliefs about the elderly, we focused on the actual treatment of the elderly across cultures. In particular, we investigated how often the elderly are included in high-power or high-status positions within each culture, while controlling for population aging. Societies with a relatively large portion of elderly people may hold positive or negative beliefs toward elderly persons (Löckenhoff et al., 2009). Thus, examining the prevalence of elderly leaders in different cultures may provide a unique lens through which to assess the respect afforded to the elderly. As an objective measure, leadership positions lend themselves well to elderly research not only because they represent the products of behaviors (i.e., appointing and electing particular people), but also because they are naturalistic (e.g., they happen in the real world, free from social desirability bias) and consequential (e.g., they have direct and significant impacts on the fate of a large group).

### Cultural Tightness, Aging, and Leadership

One important dimension on which cultures differ is *tightness-looseness* (Pelto, 1968; Triandis, 1989). Tight cultures have strong social norms and low tolerance of deviant behaviors, whereas loose cultures have weaker social norms and high tolerance of deviant behaviors (Triandis, 1989; Gelfand, 2012). Ecological, historical, and institutional factors, along with everyday situations and psychological processes, constitute and foster such distinctions of cultural systems. According to Gelfand et al. (2011), ecological and historical threats enhance the need for rigid norms and strong punishment for deviant behaviors in the society, which can help maintain social order and coordination to effectively cope with threats. Accordingly, social institutions and practices may reflect and foster cultural tightness or looseness through socialization. For example, tight cultures tend to have governing systems that suppress dissent, have media restrictions, and have strict laws. As a result, people are less likely to challenge societal institutions and norms in tight cultures than in loose cultures. Furthermore, relative to loose cultures, tight cultures place more constraints on everyday situations, which restrict the range of appropriate behaviors. All the above distal and proximal factors have an impact on individuals’ psychological processes. Thus, individuals socialized in tight cultures tend to have “self-guides that are more prevention-focused,” “are more cautious (concerned with avoiding mistakes) and dutiful (focused on behaving properly),” and “have higher self-regulatory strength… a higher need for structure, and self-monitoring ability” (Gelfand et al., 2011, p. 1101).

Uz (2015) developed three related indices for cultural tightness-looseness based on data from 68 countries in the European Values Study Group and World Values Survey Association (EWVS) integrated data set. She found traditional societies to be tighter and industrialized societies to be looser. In tighter societies, homogeneity in values, norms and behaviors was high, there were more institutional suppression, and people were less willing to live near dissimilar others.

How would cultural tightness-looseness predict the age of leaders in a culture? Tight cultures tend to value and respect tradition–an avenue to reinforce cultural norms. This claim is compatible with the positive characteristics associated with the elderly in collectivistic cultures. That is, older people are assumed to possess the key skills required to be effective leaders in tight cultures due to their extensive knowledge and practice of the social norms in a given culture. Furthermore, older people are more likely than younger people to be seen as having proven themselves through a longer “track record,” and thus choosing them as leaders may be less risky, consistent with the social and psychological practices of caution and prevention focus highlighted in tighter cultures. Researchers have also asserted that people in tighter cultures have “fewer political rights and civil liberties” (Gelfand et al., 2011, p. 1103). As a result, younger people may have fewer opportunities to get involved in leadership activities or practices.

How might cultural tightness be linked to older leadership or cultural tightness be linked to younger leadership? What could be the underlying processes in operation? One possibility is that in culturally loose systems, more diverse perspectives can proliferate (Gelfand, 2019), in turn allowing individuals to challenge the *status quo* to a greater degree. As it relates to leadership, there is no doubt that age has traditionally been associated with greater competence in several areas of leadership including wisdom (Worthy et al., 2011), maintaining stability (Spisak et al., 2014), and the ability to uphold intergroup harmony (Grossmann et al., 2010). Connecting this to the present work, being able to challenge these traditional perspectives to a greater degree may lead to individuals in loose cultures being more accepting of young leaders. Below, we outline some factors that may contribute toward tight cultures’ preference for older leaders.

First, the tightness and looseness of a culture may affect leadership preferences through the assumptions about age and experience embraced by that culture. Tight cultures are characterized by stricter social norms that are strongly enforced. This is in contrast to loose cultures, which emphasize a more open code of behavior (Gelfand, 2019). Understanding where the boundaries are located in tight cultures–and being able to use one’s lived experience to adhere to these norms–may be seen as a valuable leadership trait in this type of environment. Having accrued more lived experiences, older individuals may be seen as more knowledgeable of the strict social norms that govern tight cultures. Since enhanced knowledge has been shown to improve leader behavior and efficacy (Perkins, 2009), the experience that older leaders gain with the passage of time may give them a major competitive advantage in tight cultures.

Second, older leaders in tight cultures may not only be perceived as more knowledgeable in terms of the social norms that govern, but also may be perceived as better equipped to guide their constituents toward following these standards. Indeed, being able to maintain social order and coordination is seen as vital in tight cultures. For instance, Pelto (1968) speculated that order is required in tight cultures due to the relatively higher population density per square mile, while coordination may be imperative due to the interdependent agricultural practices. As it relates to aging, previous research has documented that older adults vary significantly from younger adults in their social motives (Imtiaz et al., 2021), especially as it relates to their preferences for familiarity and order over novelty and potential growth (Fung et al., 1999). If these preferences are projected from the individual to collective level, people may perceive older individuals who prefer order and coordination themselves to be better able to uphold this at the societal level in tight cultures. At this point, more empirical research is required to examine whether these perceptions exist among people, and how they influence leader choices across cultures.

Third, tight cultures not only endorse stricter social norms, they also enforce such norms to a higher degree by using stronger deterrents when they are violated. Thus, in order to adhere to the increased rules and regulations of tight cultures, leaders must be able to monitor and regulate their own behavior, along with the behaviors of their citizens. As such, a more cautious or preventative approach to leadership may be advantageous in this context. According to regulatory focus theory, promotion-focused individuals are motivated by gains and achievement, and are not afraid of taking potential risks on their way to successful outcomes (Higgins, 1998). In contrast, prevention-focused people view their goals as responsibilities, and prioritize risk mitigation and safety on their way to accomplishing these goals. Aging research has documented that people incline more toward a prevention focused frame of reference as they age. For instance, Micu and Chowdhury (2010) reported that older adults favored prevention focused persuasive messages, whereas younger adults showed no such preference. From a cultural perspective, Eastern societies are more prevention focused. For example, in a study examining how people pursue personal goals across cultures, Elliot et al. (2001) reported that individuals from Eastern, collectivist cultures favored a prevention focused approach (e.g., maintaining their existing social network), whereas people from Western, individualistic cultures inclined more toward a promotion focused strategy (e.g., making new friends to build their social network). All these inferences lead to our core prediction that tight (Eastern) cultures would be more likely to have older leaders compared to loose (Western) cultures.

### Present Research

In summary, the literature suggests that Eastern cultures in general hold more positive beliefs about the elderly (e.g., greater respect and adoration for elders) than do Western countries (Vauclair et al., 2017; Ackerman and Chopik, 2020). Furthermore, Eastern cultures in general tend to be tighter than Western cultures (Gelfand et al., 2011). Based on these findings, we hypothesized that older people would be more likely to hold leadership positions in Eastern than in Western countries. Furthermore, we explored whether cultural tightness would contribute to such cross-cultural differences.

We conducted two studies to test these predictions in two domains: business and political leadership. Business is a domain that is useful for examining differences in leader age across cultures, given the prevalence of globalization and international business. Political systems vary across cultures, providing a fertile ground for examining potential differences in leaders varying in age.

## Study 1

Study 1 tested whether cultures differed in the average age of their business leaders. We selected a range of countries for which we could identify ‘‘top 100 business’’ lists, identified the CEOs of these respective companies, and then determined the current age of these leaders at the time of data collection (i.e., in 2020)^2^.

### Methods

#### Observations

Based on regions and countries classified by World Economic Forum, an organization known for its authority in international business and trade^3^, we identified the two or three largest countries in population in each of the following regions: West Europe, East Europe, North Europe (Nordic), North America, South America, Middle East, East Asia, and South Asia. These regions were selected to adequately represent both Eastern and Western cultural spheres. Then, within each country, we searched online for the top 100 businesses/companies, and identified the CEO’s name and age for each company. We aimed to collect 100 leaders per country, but in practice we struggled to find data for some countries (e.g., we only identified the age of 4 Egyptian and 10 Polish leaders; see Table 1 for details). Thus, each observation consisted of a single business leader (e.g., Jose Isaac Peres of Multiplan Empreendimentos), and the leader’s current age at the time of data collection (i.e., in 2020). Additionally, the leader’s country of operation was recorded (e.g., Brazil) along with a range of country-level data (e.g., geographical region, elderly proportion, gross domestic product (GDP), GINI, cultural tightness index).

**TABLE 1:** List of included countries with region and number/ages of leaders included (Study 1).

East vs. West Description	Region	Country	# of Leaders Included	Mdn. (Mean) Leader Age	Proportion Elderly (%)	Median Age in Pop.
East	East Asia	China	88	57.0 (57.0)	11.5	38.4
		Japan	100	66.0 (67.0)	28.0	48.4
	South Asia	India	89	56.0 (55.6)	6.4	28.4
		Pakistan (We were able to find the top 40 companies in Pakistan. Among them, CEOs from 9 companies were identified, but only 2 of them had age-related information available on the internet.)	2	56.5 (56.5)	4.3	22.8
	Middle East	Egypt (We identified the top 97 companies in Egypt, and CEOs for 36 of them. Of these, only 4 CEOs had age-related information available on the internet.)	4	60.0 (59.0)	5.3	24.6
		Turkey	20	52.5 (54.6)	8.7	31.5
Other	South America	Brazil	92	52.5 (52.9)	9.3	35.2
West	North America	Canada	100	57.0 (56.7)	17.7	41.1
		United States	100	58.0 (57.6)	16.2	38.3
	Nordic	Denmark	74	55.5 (55.0)	20.0	42.3
		Sweden	90	53.0 (52.7)	9.1	41.1
	West Europe	Germany	104	55.0 (53.9)	21.6	45.7
		United Kingdom	101	54.0 (55.1)	18.5	40.5
	East Europe	Poland (We were able to identify 19 companies and their CEOs in Poland. Of these, only 10 had age-related information available on the internet.)	10	55.0 (52.4)	18.1	41.7
		Russia	60	54.0 (53.9)	15.1	39.6
–	Total/Median of Countries	–	1034	56.0 (56.2)	15.1	39.6

#### Measures

##### Economic indices

We drew the most recent available GDP-per-capita and GINI data from the World Bank (2019)^4^. GDP-per-capita was assessed in United States dollars and can be interpreted as a measure of economic productivity or approximate wealth. We calculated log-scores for GDP to counterbalance the skewed distribution of GDP scores. The GINI index assesses economic dispersion (i.e., higher GINI scores indicate that fewer people hold a greater proportion of wealth), and it has a conceptual range of 0–100 (0 = perfect equality, 100 = perfect inequality). These were added as covariates to help verify that culture-irrelevant differences in economic thriving did not account for our effects.

##### Cultural tightness

Cultural tightness scores were drawn from Uz (2015). We used the “CTL_C” measure. Cultural tightness is associated across multiple domains: work, political, religious, and family. The weighting of each domain is determined by how important people in that country see the domain as being (for a comprehensive explanation, see Uz, 2015). Because higher CTL_C scores represent less tightness, we reversed the scale so that higher scores could indicate increased tightness (*M* = 51.7, *SD* = 24.0).

##### Elderly proportion

Scores were obtained from the World Bank and represent the percentage of the population that is 65+ (i.e., a common cut-off for being a senior), and thus has a conceptual range of 0–100^5^.

### Results

#### Cultural Variance in Leader Age

**FIGURE 1:**
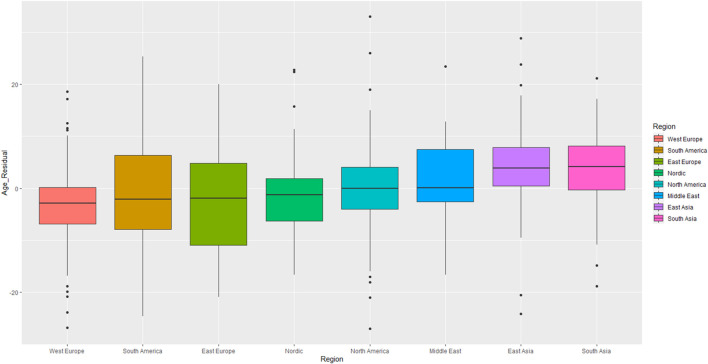
Business leader ages by geographical region. Age depicted in the figure is adjusted for the presence of the economic and elderly population covariates. See SOM for figures presenting raw (i.e., unadjusted by covariate) age scores.

We began by testing if average leader age varied by country, using ANCOVA models in which the broad regions to which countries could be assigned were set as the predictor variable (i.e., North America, East Europe, West Europe, Nordic, South America, Middle East, East Asia, and South Asia). The covariate was elderly proportion. This produced a main effect of region, *F*(7, 1025) = 20.21, *p* < 0.001, η^2^*_*p*_* = 0.12; leaders’ average age varied by region. Figure 1 displays boxplots of leader ages for each region, arranged left to right from youngest to oldest. European states are the youngest, whereas East/South Asian states are the oldest. In the same model, we found a main effect of elderly proportion, *F*(1, 1025) = 68.70, *p* < 0.001, η^2^*_*p*_* = 0.06, such that leaders who lived in areas with more elderly tended to be more elderly themselves.

**FIGURE 2:**
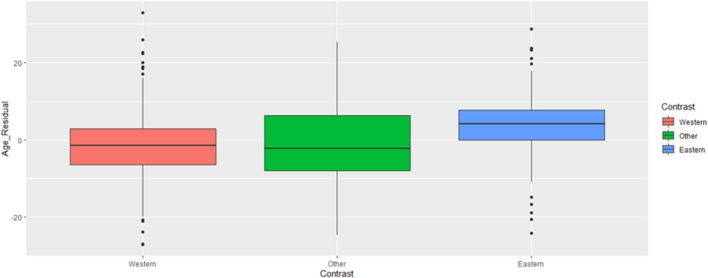
Business leader ages by culture contrast. Age depicted in the figure is adjusted for the presence of the economic and elderly population covariates. See SOM for figures presenting raw (i.e., unadjusted by covariate) age scores.

To test our key prediction, we created a contrast variable such that Western cultures (including West/East/North Europe and North America) were scored −0.5, Eastern cultures (East/South Asia and Middle East) were scored +0.5, and other cultures (South America) were scored 0. The logic of this analysis is that it weighs the cultures such that Eastern cultures are being compared against Western cultures, positive effects of the contrast indicating that Eastern cultures are associated with more of a variable. We then regressed leader age on this contrast variable and elderly population proportion. Our contrast term was supported by the data, *B* = 5.48 [4.43, 6.54], *t*(1031) = 10.22, *p* < 0.001. Specifically, Western countries (*M*_adj_ = 54.5, *SE* = 0.31) had the youngest leaders, Eastern countries had the oldest leaders (*M*_adj_ = 60.0, *SE* = 0.44), with other countries falling between (*M*_adj_ = 55.7, *SE* = 0.84). This is also mirrored in the plot provided as Figure 2. Broadly, the plot indicates that the Western (red) countries generally had younger leaders, and Eastern countries (blue) had older leaders. Generally, the other (green; non-Western/Eastern) countries resembled the Western more than the Eastern range. We also replicated the effect whereby older leaders emerged in countries with larger elderly populations, *B* = 0.46 [0.39, 0.54], *t*(1031) = 11.92, *p* < 0.001^6^.

#### Preregistered Linear Modeling Tests

Next we proceeded to a series of follow-up analyses intended to better understand the culture-based age effect^7^. As we expected, adjusting for GDP and GINI did nothing to change the effect of the cultural contrast term, and only GDP related to leader age, *B* = 1.22 [0.77, 1.67], *t*(1029) = 5.32, *p* < 0.001. Our cultural contrast term remained significant, *B* = 6.67 [5.52, 7.81], *t*(1029) = 11.45, *p* < 0.001, as did the elderly proportion effect, *B* = 0.39 [0.30, 0.47], *t*(1029) = 8.85, *p* < 0.001.

Following the preregistration, we checked for mediation using Hayes’s (2017) PROCESS Model 4, and the indirect effect was non-significant, *IE* = 0.35 [−1.21, 1.92]. Importantly, the *a*-path from culture contrast to cultural tightness was significantly positive, *B* = 33.58 [31.60, 35.57], *t*(748) = 33.20, *p* < 0.0001^8^. Indeed, this effect indicates that Eastern cultures were culturally tighter than Western ones, consistent with our theorizing. However, the *b*-path between cultural tightness and leader age was non-significant, *B* = 0.01 [−0.02, 0.05], *t*(747) = 0.46, *p* = 0.647.

### Discussion

Study 1 provided some support for our hypotheses. First, we found significant cross-region heterogeneity in business leaders’ ages, such that business leaders tend to be older in Eastern than Western countries. Thus, preferences for older leaders are not entirely universal, and may be related to individual characteristics of cultures. Neither population structure (elderly proportion) nor economic factors (GDP, GINI) explained away the effect. Finally, we found that Eastern cultures were culturally tighter than Western cultures.

We did not find significant support for cultural tightness connecting with leader age. One possible reason for this is that we did not have a sufficient sample size to be powered to detect this pattern, as only a subset of our assessed cultures (11 out of 15) had cultural tightness scores available. Thus, our relatively large sample size shrunk substantially for the mechanistic analysis, which might have led to a Type II error for this analysis.

A second possibility is that business leaders may not be as susceptible to cultural influences as other leaders (e.g., political leaders). That is, high-level business management could cultivate a meritocratic environment wherein cultural preferences have less influence. As such, a stronger case might be made by examining leaders who generally are appointed by popular vote, and therefore might be more susceptible to cultural beliefs and values held by the broad public. Hence, in Study 2 we looked at political leaders.

## Study 2

The main goal of Study 2 was to replicate Study 1 in a different (political) domain. We identified the previous five political leaders for all the countries in the world (if information was available), and then compared the age of these leaders. We then examined a range of cultural variables (including cultural tightness) as potential mechanisms accounting for the cultural heterogeneity in political leaders’ age.

### Methods

#### Observations

Each observation consisted of a single political leader (e.g., Cyril Ramaphosa), the leader’s age at the commencement of his/her appointment, the leader’s country of operation (e.g., South Africa), along with a range of country-level data (e.g., elderly proportion, GDP, GINI, Hofstede culture-level values, cultural tightness index). We aimed to collect at least five leaders per country, and were able to get the information from 191 countries (out of the total 195 countries in the world). Some countries had more than one major political leader (e.g., India has both a prime minister and a president), in which case we recorded up to ten leaders (e.g., both the last five prime ministers and the last five presidents). Some countries placed the same individual in power more than once (e.g., Sheikh Hasina of Bangladesh) in which case this leader was used multiple times, with their age recorded at each commencement of appointment.

#### Measures

Measures remained from Study 1, with one addition explained below.

##### World value survey questions

The World Value Data was taken from Wave 7 (2017–2021). Specific item selections are explained in the preregistration document, but we assessed clusters of items that attempted to assess distinct constructs. We selected items related to gender beliefs (six items), innovation beliefs (six items), distancing from stigmatized groups (seven items), elderly veneration (two items), and left/right political orientation (one item). We then used a series of factor analyses to determine how many factors best captured these item batteries. We identified two factors for gender beliefs: one relating to *prioritizing male leadership* (in politics, university, business), and the other relating to *prioritizing men’s wages* (under scarcity, as compared to women). We found two factors for innovation beliefs: one relating to *utilitarian science benefits* (making life better, more opportunities, world better off), and the other relating to *science and core values* (science vs. faith, science undermines morals, science irrelevant to personal life). We found two factors relating to intolerance: *distancing from sexually stigmatized groups* (AIDS and gay people), and *distancing from foreign culture* (race, immigrants, other religions, speaking other languages). For elderly veneration norms there were only two items, and these correlated highly to represent a single index of *venerating parents*. These constructs were selected to help us understand the mechanisms responsible for cultural differences in leaders’ age.

### Results

#### Cultural Variance in Leader Age

**FIGURE 3:**
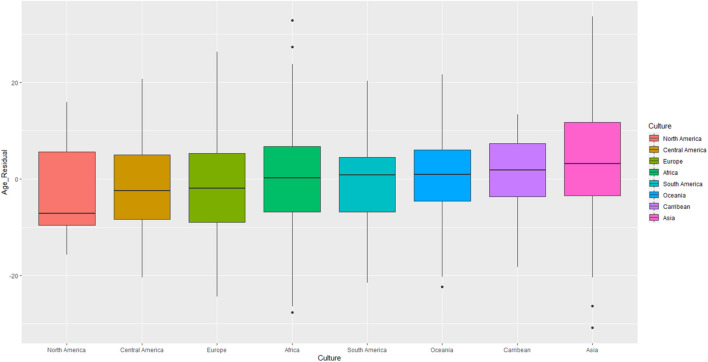
Political leader ages by geographical region (Study 2). Age depicted in the figure is adjusted for the presence of the economic and elderly population covariates. See SOM for figures presenting raw (i.e., unadjusted by covariate) age scores.

We tested if political leaders’ ages differed by region using ANCOVA models in which the broad regions to which countries could be assigned were set as the predictor variable (i.e., North America, Central America, South America, Caribbean, Europe, Asia, Africa, and Oceania)^9^. This produced a main effect of region, *F*(7, 1191) = 7.74, *p* < 0.001, η^2^*_*p*_* = 0.04, indicating that leaders’ average age differed significantly by region. Figure 3 displays distinct boxplots of leader ages for each geographical region. North America had the youngest leaders, followed by Central America and Europe. In contrast, the oldest leaders were found in Caribbean and Eastern countries. In the same model, we found a main effect of elderly proportion, *F*(1, 1191) = 18.24, *p* < 0.001, η^2^*_*p*_* = 0.02, such that regions with more elderly tended to have older leaders^10^.

**FIGURE 4:**
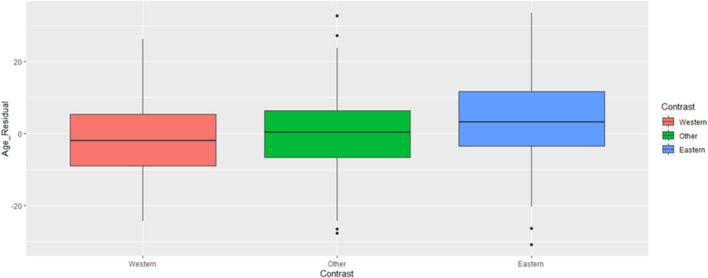
Political leader ages by culture contrast (Study 2). Age depicted in the figure is adjusted for the presence of the economic and elderly population covariates. See SOM for figures presenting raw (i.e., unadjusted by covariate) age scores.

To better understand the effect of region, we created a contrast variable as in Study 1 (Western cultures including Europe and North America = −0.5, Eastern cultures including East/South Asian and Middle Eastern = +0.5, other cultures = 0)^11^. We then used this contrast variable and elderly population proportion to predict leader age. Our contrast term was supported by the data, *B* = 6.91 [4.63, 9.19], *t*(1197) = 5.94, *p* < 0.001. Specifically, Western countries (*M*_adj_ = 49.26, *SE* = 0.88) had the youngest leaders, Eastern countries had older leaders (*M*_adj_ = 58.21, *SE* = 0.72), and other countries (*M*_adj_ = 57.15, *SE* = 0.55) fell between. Figure 4 displays these differences. Western countries had the youngest leaders, Eastern the oldest. Unsurprisingly, we replicated the effect whereby older leaders tended to emerge in countries with larger elderly populations, *B* = 0.16 [0.04, 0.28], *t*(1197) = 2.55, *p* = 0.011^12^.

#### Preregistered Linear Modeling Tests

Next we proceeded to a series of follow-up analyses intended to better understand the culture-based age effect^13^. As expected, adjusting for GDP and GINI did not change the effect of the cultural contrast test, which remained significant, *B* = 9.67 [7.25, 12.09], *t*(1055) = 7.85, *p* < 0.001, as did the elderly population effect, *B* = 0.20 [0.01, 0.39], *t*(1055) = 2.07, *p* = 0.038. We found a marginal effect of GDP, *B* = 0.64 [−0.06, 1.34], *t*(1055) = 1.79, *p* = 0.074, and a significant effect of GINI, *B* = 0.16 [0.08, 0.25], *t*(1055) = 3.62, *p* < 0.001. These effects suggested that more economically unequal countries, and possibly more economically advantaged countries, tended to have slightly older political leaders.

Next, we analyzed possible indirect effects using PROCESS model 4 (Hayes, 2017)^14^. Each mechanism was tested in a separate mediation analysis, reflected in the rows of Table 2. Effects were non-significant for most mechanism variables. Note that most mechanism variables were significantly related to culture, as indicated by the statistically significant *a*-paths. That is, compared to Western countries, Eastern countries tended to prioritize male over female leadership and wages, valued the utilitarian benefits of science more (with less belief that science undermines morality), venerated the elderly more, had more desire to be distanced both from sexually stigmatized groups and from cultural minority groups, and were culturally tighter.

**TABLE 2:** Indirect effects from culture contrast to political leader age (Study 2).

Mediator	*a*-path (culture contrast to mediator)	*b*-path (mediator to leader age)	Indirect effect (*a* × *b*)	Direct effect	*n* for analysis
Prioritizing Male Leadership	0.39***	–0.68	−0.27 [−2.19, 1.60]	6.07 [1.94, 10.20]	261
Prioritizing Male Wages	0.34***	2.48	0.85 [−0.31, 2.02]	4.96 [1.13, 8.79]	261
Science/Utilitarianism	0.44***	–1.09	−0.48 [−1.52, 0.50]	6.29 [2.50, 10.07]	261
Science/Core Values	−0.49***	0.97	−0.47 [−1.43, 0.43]	6.28 [2.49, 10.07]	261
Veneration of Elderly	0.24***	2.37	0.57 [−0.23, 1.40]	5.23 [1.48, 8.99]	261
Distance/Sexually Stigmatized	0.25***	3.18	0.81 [−0.48, 2.15]	5.68 [1.64, 9.72]	249
Distance/Culture	0.07***	8.44	0.63 [0.00, 1.45]	5.18 [1.45, 8.91]	261
Cultural Tightness	16.31***	0.06**	1.02 [0.28, 1.84]	9.00 [5.75, 12.25]	440

**p < 0.01, ***p < 0.001.

*Values in square brackets refer to 95% confidence intervals.*

However, most *b*-paths were non-significant^15^, and indeed only one cultural variable, cultural tightness, was significantly connected to older leadership. Specifically, countries that were culturally tighter had significantly older leaders, *B* = 0.06 [0.02, 0.11], *t*(436) = 2.75, *p* = 0.006. A statistically significant indirect effect from culture to political leader age through cultural tightness was identified. Thus, Eastern (vs. Western) cultures tend to be culturally tighter, and tighter cultures have older political leaders. The reported indirect effect remained significant when adjusting for the economic variables, *IE* = 1.72 [0.79, 2.81].

### Discussion

Study 2 provides a clear conceptual replication of Study 1, demonstrating significant cultural heterogeneity in the ages of political leaders. In particular, Eastern leaders were older than Western leaders. The effects remained significant when controlling for population structure and economic factors. Furthermore, Study 2 identified a possible cultural mechanism: as hypothesized, Eastern cultures tend to be culturally tighter, which mediated cultural differences in political leaders’ age.

## General Discussion

### Summary of Findings

The present work aimed to provide a new perspective on aging and leadership across cultures by providing a simple test: what types of cultures tend to have older vs. younger leaders? Study 1 reported that business leaders (e.g., CEO’s of major corporations) were older in Eastern countries compared to their peers in Western nations, even while controlling for percentage of elderly in the society, GDP, and GINI. Study 2 conceptually replicated these findings by illustrating that political leaders (e.g., presidents, prime ministers) were once again older in Eastern countries compared to Western ones, even while adjusting for the percentage of the population that is elderly, GDP, and GINI. Further, we found that cultural tightness accounted for these patterns. That is, Eastern cultures were more culturally tight than Western cultures, and cultural tightness, in turn, predicted having older leaders.

### Implications of Current Findings

The present results shed some light on an ongoing discussion about cultural differences in how elderly individuals are viewed (Löckenhoff et al., 2009; Vauclair et al., 2017; Ackerman and Chopik, 2020). Beyond economic (e.g., GDP) and demographic features (e.g., percentage elderly population), culture plays a role in how likely elderly people are to assume high-power business or political positions. Of course, this finding is distinct from attitudes toward the elderly, as most prior research has focused on. We suggest that a culture’s tendency to facilitate/inhibit a social group to occupy high-power roles is important above and beyond positive/negative evaluations made about that group within those cultures. Furthermore, Study 2 revealed a specific cultural variable–tightness/looseness (Uz, 2015)–that accounted for part of this Western/Eastern difference. This helps to establish cultural tightness as a key cultural factor by demonstrating that it can account for a high-stakes phenomenon across societies: the tendency to have elderly people gain or maintain authority.

Differences in leader age across cultures may have a significant impact on how these individuals interact and negotiate with one another on the international stage. For example, as new heads of state interact with one another for the first time, generational differences may create friction if older leaders from Eastern countries have difficulty finding common ground with younger leaders from Western countries. For instance, past research has documented how leader age has a significant impact on foreign policy, including one’s willingness to escalate military disputes. Indeed, in a longitudinal study examining interactions between global leaders during 1875–2002, Horowitz et al. (2005) found that older leaders were more likely to initiate and intensify military conflicts compared to their younger peers. These findings highlight how subtle differences, some of which may go unnoticed when examining geopolitical issues, have the potential to have major implications on critical issues such as war. It is important to note that these issues may not be exclusive to political interactions. Indeed, business leaders of international companies may also have similar problems as they negotiate at the international level in an increasingly globalized business landscape.

Beyond the issues that may arise between international leaders from varying cultures, group dynamics within multi-generational teams are also important to consider as globalization continues to make cross-culture interactions more frequent. For instance, research has documented that age differences between leaders and their teams have the potential to produce significant friction at the on-set of the relationship (Harrison et al., 2002). Thus, Eastern leaders interacting with Western subordinates (or vice versa) may experience significant challenges in building rapport if their subordinates are used to interacting with relatively younger leaders.

Along the same lines, acculturation research has documented that diverse work teams composed of individuals from varying cultures will become increasingly prevalent in tomorrow’s business world (Luijters et al., 2006). Unlike the leader-subordinate relationship, which may be characterized by important but few interactions, peer-to-peer diversity on work teams has the potential to be even more impactful on an organization’s daily functions. Related to the present work, if individuals on diverse work teams have different views on what their leaders expect and how they should interact with them, this may negatively impact group dynamics within their teams as well as how they approach their work.

Lastly, international companies (e.g., HSBC, Google, and Amazon) operating in today’s globalized world do not rely on one sole leader, but often numerous directors to lead their various branches around the world. If these organizations hire leaders based on the preferences of their home cultures, this may lead to issues if these leaders are rejected in the cultures of their satellite branches. For example, if a Western company hires a relatively young leader to lead a team located in an Eastern part of the world, this individual may face backlash from employees who are used to, and prefer older leaders. Related to this, past research has shown that adapting organizational values to a host culture is critical for ensuring international business success. A well-documented example of this was the closure of a Starbucks café in China’s Forbidden City–one of the most important cultural sites in Beijing (Han and Zhang, 2009). One of the main lessons from this case study was that global brands need to be careful and deliberate when expanding beyond their home cultures so that they are not perceived as infringing on the culture and history of other cultures. In the same way, organizations may benefit from being intentional when determining the type of leader that they want to appoint in international markets outside of their home cultures.

### Limitations and Future Directions

We recognize that the present findings are based on correlational data. As a result, we cannot rule out an alternative causal chain: that Eastern cultures tend to appoint or support older leaders, and older leaders tend to establish increased cultural tightness through the sorts of policies that they support. Indeed, cultural tightness and older leaders may be mutually reinforcing, with each variable causing changes in the other over time. Statistical analyses based on correlational evidence cannot determine causality or its direction (see Thoemmes, 2015; Lemmer and Gollwitzer, 2017). Usually, experimental designs are employed to clarify causality between variables (Spencer et al., 2005). For instance, cultural priming (Hong et al., 2000) may be utilized to experimentally test whether adopting a particular cultural frame influences leader age preference. In addition, longitudinal designs may help reveal if cultural tightness is responsible for preferences for older leaders.

Another limitation of the current work is that it examined leader age in two specific contexts (i.e., politics and business). Thus, establishing the generalizability of the present findings by exploring novel contexts will be an important undertaking for future research. Even within the same culture, it may be that differences in leader age emerge across unique sectors (e.g., banking; tech start-ups; shared economy). Along the same lines, within a given country, several regional cultures may emerge. As such, would one expect differences in leader age across these distinct intra-country cultures?

Related to this, future research should investigate whether the hierarchal nature of the domain being studied would moderate leader age preferences across cultures. That is, domains that are characterized by relatively strong vertical hierarchies (e.g., the military; academia) may be more immune to age differences across cultures due to the significant amount of time and experience that it takes to rise in the leadership ranks. In contrast, less hierarchal domains (e.g., politics; business; sport) – where popularity often determines leadership positions–may be more heavily influenced by cultural preferences as it relates to leader age. The impact of cultural tightness and looseness on these relationships also requires further exploration in future research.

Another limitation in the present work was that the cultural tightness mediation effect was only present in Study 2. This may have been due to the relatively small number of countries for this analysis in Study 1. Alternatively, the lack of mediation may be due to context, as business practices may be fundamentally different than those in politics. For instance, unlike politics, where the perceptions of the masses often dictates leader choice, many business contexts are not as reliant on wide scale preferences. Instead, leadership positions in business are often determined by a select group of individuals, or handed down generationally as is the case in family run organizations. Thus, even though the main effect of leader age across cultures may hold across politics and business in general, the mediation by cultural tightness may not be as impactful in business as it is in politics.

Another interesting avenue for future research involves the impact of aging societies around the world. Indeed, it is well established that most societies around the world are aging rapidly, and this effect is perhaps most pronounced in highly developed Western countries (Vincent and Velkoff, 2010; Harper, 2014). As Western leaders become older as a by-product of these demographic shifts, it will be interesting to see whether they remain relatively younger compared to leaders in the East. That is, will the pace at which Western countries are aging eliminate the findings reported in the present work, or will this be compensated for by the universal aging trends around the world? Further, will aging Western countries become tighter culturally because of being led and governed by older leaders over time? To examine this final question, longitudinal data will be required to test how societal aging trends shape leader age around the world.

One final path for future research to explore involves the role of pathogen theory on cultural preferences for older and younger leaders. Previous research has outlined that the prevalence of pathogens influences societal orientations. For example, Jackson et al. (2020) recently reported that cultural tightness was positively correlated with pathogen prevalence across a large-scale study spanning 86 non-industrialized societies. The researchers asserted that cultural tightness may be advantageous during times of pathogen prevalence as strong social norms aimed at mitigating pathogen transmission and harsher punishments for breaking those norms may deter future outbreaks. As it relates to the current work, future research should investigate whether this relationship has any bearing on preferences for older leaders, who were found to be more prevalent in tight cultures. This line of work may be especially interesting to pursue considering the recent global Covid-19 pandemic.

## Conclusion

In sum, the present research has shown that business and political leaders tend to be older in Eastern countries than in Western countries. Cultural tightness seems to play an important role in such effects. That is, Eastern countries are culturally tighter, and cultural tightness positively predicted the presence of older leaders. Future research should examine the possible causal links underlying the cultural effect on leader age, and explore various practical implications of the effect.

## Data Availability Statement

The raw data supporting the conclusions of this article will be made available by the authors, without undue reservation.

## Author Contributions

All authors conceived the research idea, designed the studies, did the literature review, wrote the manuscript, undertook final clarification, and agreed on the version of the manuscript for submission. L-JJ collected the data. TV-J analyzed the data.

## Conflict of Interest

The authors declare that the research was conducted in the absence of any commercial or financial relationships that could be construed as a potential conflict of interest.

## Publisher’s Note

All claims expressed in this article are solely those of the authors and do not necessarily represent those of their affiliated organizations, or those of the publisher, the editors and the reviewers. Any product that may be evaluated in this article, or claim that may be made by its manufacturer, is not guaranteed or endorsed by the publisher.

## Footnotes

1 Note some of the measures may be problematic. For example, although Chinese or Taiwanese participants have positive views about the elderly, making friends with them or spending the whole day with them (measure of indirect prejudice) may not be consistent with the cultural norms. To many Chinese, the elderly are to be respected, not to be befriended.

2 A careful reader might wonder why we did not collect the age when the leader was appointed, rather than their present age. From our perspective, either measure would be valid. Business leaders not only have to become CEOs but also hold these positions across time, requiring the ongoing support of shareholders and executive officers; thus, their age at a random moment in time is meaningful, just as their age at the time of appointment would be meaningful. Additionally, it was often difficult to ascertain the leader’s age at the time of their original CEO appointment.

3
http://reports.weforum.org/

4
https://data.worldbank.org

5 Relatedly, a reviewer recommended we also consider median age (from United Nations, 2019); however, we found that this correlated with elderly proportion at *r*(1191) = 0.87, *p* < 0.001, so we did not include median age in the main text analyses. If median age is substituted for elderly proportion as a covariate, or both age-related covariates added together, the pattern of effects remains unchanged.

6 Similar patterns of results were detected in a model without covariates. The effect remained significant using raw (unadjusted by covariate) age scores, as supported by a significant ANOVA across regions, *F*(7, 1026) = 24.39, *p* < 0.001, and significant planned contrast, *B* = 4.38 [3.27, 5.48], *t*(1032) = 7.77, *p* < 0.001.

7 The preregistration document is available at https://osf.io/bp8vq/?view_only=9498b31b38394928aeae5c69e29fe40d. In both studies we ran some analyses as regressions rather than ANCOVAs to closer match the mediation analyses, but results remain very similar in ANCOVA.

8 Note that the denominator degrees of freedom dropped in these tests compared to the previous paragraph because not all countries had cultural tightness scores.

9 These regions (based on https://www.dhs.gov/geographic-regions) differed from Study 1. They were used instead to test the robustness of the expected results. We expected older leaders in Eastern than in Western countries regardless of the source on which the groupings of countries were based.

10 Again we considered each country’s median age as a covariate, but its very high correlation with elderly proportion, *r*(1220) = 0.95, *p* < 0.001, led us to dismiss it as an additional covariate.

11 The presented analyses assign Oceania to be “Other,” but results remain very similar if Oceania is set as Western (e.g., see Leung et al., 2011). For example, the main effect of cultural contrast on leader age (controlling for proportion elderly) remains significant, *F*(2, 1196) = 16.80, *p* < 0.001, η^2^*_*p*_* = 0.03 even with Oceania states set as Western.

12 Dropping the covariate nonetheless reveals a significant omnibus test across regions, *F*(7, 1260) = 6.44, *p* < 0.001, and a main effect of our contrast, *B* = 5.44 [3.74, 7.15], *t*(1266) = 6.27, *p* < 0.001.

13 The registration document is available at https://osf.io/c7fub/?view_only=14a1e2ad234749cfad54ded409a83c5a and https://osf.io/wpf6v/?view_only=1aa43b25addb46e7a797576ba78fcd2d. Although we preregistered running these analyses in multilevel modeling (and did), linear modeling revealed very similar effects so we focus on linear tests instead for the sake of simplicity.

14 Unfortunately, our preregistration’s proposal to assess if the effects of the cultural contrast variable dropped when including each cultural mechanism variable proved fruitless. This was because of the broad degree of missing data for these variables, which meant that comparing models before/after the inclusion of the cultural variables was difficult to interpret.

15 There was a significant indirect effect from culture to political leader age through ostracism of stigmatized cultures. The a-path from culture to ostracism was significant; however, the *b*-path from ostracism to leader age was non-significant, *B* = 8.44 [−1.17, 18.05], *t*(257) = 1.73, *p* = 0.085. Thus, although this test met Hayes’s (2017) index test for mediation, it failed to meet joint-significance test standards (Yzerbyt et al., 2018), so we do not discuss it further.
