# Corrigendum: Age differences in leadership positions across cultures

**DOI:** 10.3389/fpsyg.2023.1129019

**Published:** 2023-02-07

**Authors:** Thomas Vaughan-Johnston, Faizan Imtiaz, Albert Lee, Li-Jun Ji

**Affiliations:** ^1^Department of Psychology, Queen's University, Kingston, ON, Canada; ^2^Department of Psychology, Durham University, Durham, United Kingdom; ^3^Department of Psychology, Towson University, Towson, MD, United States; ^4^School of Social Sciences, Nanyang Technological University, Singapore, Singapore

**Keywords:** leadership, aging, culture, cultural tightness, business, politics

In the published article, there was an error in Table 2. For “Prioritizing Male Leadership,” the *a*-path was stated as 0.70^***^, *b*-path as −0.84, indirect effect as −0.59 [-1.73, 0.50], and direct effect as 6.39 [2.56, 10.22]; those values are corrected to 0.39^***^, −0.68, −0.27 [-2.19, 1.60], and 6.07 [1.94, 10.20], respectively. For “Prioritizing Male Wages,” the *a*-path/*b*-path were stated as −0.34^***^/-2.48, but are corrected to 0.34^***^/2.48, respectively. For “Veneration of Elderly,” the *a*-path/*b*-path were stated as −0.24^***^/-2.37, but are corrected to 0.24^***^/2.37, respectively. The corrected Table 2 and its caption appear below.

**Table 2:** Indirect effects from culture contrast to political leader age (Study 2).

**Mediator**	***a*-path (culture contrast to mediator)**	***b*-path (mediator to leader age)**	**Indirect effect (*a* X *b*)**	**Direct effect**	***n* for analysis**
Prioritizing Male Leadership	0.39^***^	−0.68	−0.27 [-2.19, 1.60]	6.07 [1.94, 10.20]	261
Prioritizing Male Wages	0.34^***^	2.48	0.85 [-0.31, 2.02]	4.96 [1.13, 8.79]	261
Science/Utilitarianism	0.44^***^	−1.09	−0.48 [-1.52, 0.50]	6.29 [2.50, 10.07]	261
Science/Core Values	−0.49^***^	0.97	−0.47 [-1.43, 0.43]	6.28 [2.49, 10.07]	261
Veneration of Elderly	0.24^***^	2.37	0.57 [-0.23, 1.40]	5.23 [1.48, 8.99]	261
Distance/Sexually Stigmatized	0.25^***^	3.18	0.81 [-0.48, 2.15]	5.68 [1.64, 9.72]	249
Distance/Culture	0.07^***^	8.44	0.63 [0.00, 1.45]	5.18 [1.45, 8.91]	261
Cultural Tightness	16.31^***^	0.06^**^	1.02 [0.28, 1.84]	9.00 [5.75, 12.25]	440

^**^p < 0.01, ^***^p < 0.001.

Values in square brackets refer to 95% confidence intervals.

In the published article, there was also an error in the text. A correction has been made to **Results**, *Preregistered Linear Modeling Tests*, paragraph 2. This sentence previously stated:

“That is, compared to Western countries, Eastern countries tended to prioritize male over female leadership (but male wages less), valued the utilitarian benefits of science more (with less belief that science undermines morality), venerated the elderly less, had more desire to be distanced both from sexually stigmatized groups and from cultural minority groups, and were culturally tighter.”

The corrected sentence appears below:

“That is, compared to Western countries, Eastern countries tended to prioritize male over female leadership and wages, valued the utilitarian benefits of science more (with less belief that science undermines morality), venerated the elderly more, had more desire to be distanced both from sexually stigmatized groups and from cultural minority groups, and were culturally tighter.”

The authors apologize for this error and state that it does not change the primary scientific conclusions of the article. The original article has been updated.

