# Supplementary material for: Age Differences in Leadership Positions Across Cultures
Source: Front Psychol. 2021 Sep 17;12:703831. doi: 10.3389/fpsyg.2021.703831 (PMC8484309; doi:10.3389/fpsyg.2021.703831)

Supplementary Online Materials

Table S-1. *Average Age of Leaders in Individual Countries.*

Country	Age	Country	Age	Country	Age	Country	Age	Country	Age
1. North Korea	28.0	39. Sweden	49.2	77. Egypt	52.6	115. Dominica	56.3	154. Greece	60.4
2. Oman	33.4	40. Cambodia	49.4	78. Seychelles	52.6	116. Argentina	56.4	155. Iraq	60.4
3. Brunei	35.4	41. Ethiopia	49.5	79. Timor-Leste	52.6	117. Indonesia	56.4	156. Zimbabwe	60.5
4. Chad	39.0	42. Turkmenistan	49.5	80. Dominican Republic	52.8	118. Ivory Coast	56.5	157. Turkey	60.8
5. Liechtenstein	39.8	43. Belarus	49.8	81. Croatia	53.1	119. Yemen	56.5	158. Lebanon	60.9
6. Nauru	43.0	44. Andorra	50.0	82. Portugal	53.1	120. Guyana	56.6	159. Guatemala	61.0
7. San Marino	43.9	45. Serbia	50.0	83. Niger	53.2	121. Central African Republic	56.8	160. Zambia	61.0
8. Burundi	45.1	46. Sierra Leone	50.0	84. Cuba	53.2	122. St. Vincent & Grenadines	56.8	161. Chile	61.2
9. Denmark	45.6	47. Djibouti	50.1	85. Tajikistan	53.3	123. Nicaragua	56.8	162. Mali	61.3
10. Spain	45.6	48. Barbados	50.2	86. Germany	53.4	124. Sao Tome and Principe	56.8	163. Trinidad and Tobago	61.3
11. Gambia	45.8	49. Maldives	50.2	87. Jamaica	53.4	125. Solomon Islands	56.8	164. Jordan	61.5
12. Syria	46.0	50. Slovenia	50.2	88. Panama	53.4	126. Uganda	56.8	165. Japan	61.6
13. Austria	46.2	51. Cabo Verde	50.3	89. Australia	53.6	127. Guinea	56.9	166. Monaco	62.2
14. North Macedonia	46.3	52. UAE	50.3	90. Botswana	53.8	128. Antigua and Barbuda	57.0	167. Italy	62.8
15. Saint Kitts and Nevis	46.3	53. Iceland	50.4	91. France	54.1	129. Comoros	57.0	168. Vietnam	63.0
16. Netherlands	46.4	54. Kyrgyzstan	50.5	92. Benin	54.2	130. Samoa	57.0	169. Morocco	63.2
17. New Zealand	46.4	55. Togo	50.6	93. Cameroon	54.3	131. Switzerland	57.6	170. Nepal	63.3
18. El Salvador	46.6	56. Estonia	50.7	94. Tanzania	54.3	132. Haiti	57.8	171. Peru	63.4
19. Montenegro	46.8	57. Bolivia	50.8	95. Hungary	54.5	133. Azerbaijan	57.8	172. South Korea	63.5
20. Belgium	46.8	58. Angola	51.0	96. Lithuania	54.6	134. Bosnia and Herzegovina	58.0	173. Algeria	63.6
21. Qatar	46.9	59. Paraguay	51.0	97. Madagascar	54.6	135. Saint Lucia	58.0	174. Namibia	63.9
22. Eritrea	47.0	60. Poland	51.0	98. Mauritania	54.7	136. Tonga	58.0	175. Brazil	64.2
23. Ukraine	47.0	61. South Sudan	51.0	99. Gabon	54.7	137. Vanuatu	58.2	176. Israel	64.4
24. Rwanda	47.2	62. Mexico	51.2	100. Bhutan	55.0	138. Grenada	58.2	177. Malawi	64.4
25. Kiribati	47.2	63. Papua New Guinea	51.2	101. Romania	55.0	139. Tunisia	58.3	178. Myanmar	64.4
26. French Guiana	47.3	64. Mozambique	51.2	102. Thailand	55.0	140. Bahamas	58.6	179. Kuwait	64.6
27. Colombia	47.4	65. Slovakia	51.5	103. Guine-Bissau	55.2	141. Belize	58.6	180. South Africa	64.8
28. Luxembourg	47.4	66. Finland	51.6	104. Somalia	55.2	142. Eswatini	58.6	181. Fiji	64.8
29. Afghanistan	47.5	67. Canada	51.8	105. Czech Republic	55.6	143. Marshall Islands	58.9	182. Mauritius	64.9
30. Georgia	47.6	68. DR Congo	51.9	106. Uzbekistan	55.7	144. Venezuela	59.2	183. Cyprus	65.0
31. Kosovo	47.7	69. Kazakhstan	51.9	107. Latvia	55.7	145. Malta	59.2	184. Uruguay	65.0
32. Sudan	47.8	70. Armenia	51.9	108. Senegal	55.8	146. Northern Ireland	59.3	185. Sri Lanka	66.6
33. Mongolia	47.9	71. Russia	52.0	109. Costa Rica	55.8	147. Nigeria	59.4	186. Bangladesh	67.6
34. Bulgaria	47.9	72. Congo	52.1	110. Honduras	55.8	149. Suriname	59.6	187. China	67.6
35. Bahrain	48.0	73. Iran	52.2	111. Ecuador	56.0	150. Philippines	59.8	188. Malaysia	67.6
36. Norway	48.6	74. Burkina Faso	52.3	112. Liberia	56.0	151. Singapore	59.9	189. Lesotho	68.0
37. Moldova	49.0	75. United Kingdom	52.4	113. Tuvalu	56.1	152. Ghana	60.0	190. India	71.2
38. Albania	49.1	76. Palau	52.5	114. United States	56.2	153. Libya	60.0	191. Laos	75.0

Figure S-1.

Business Leader Ages (Raw) by Geographical Region. (Study 1).

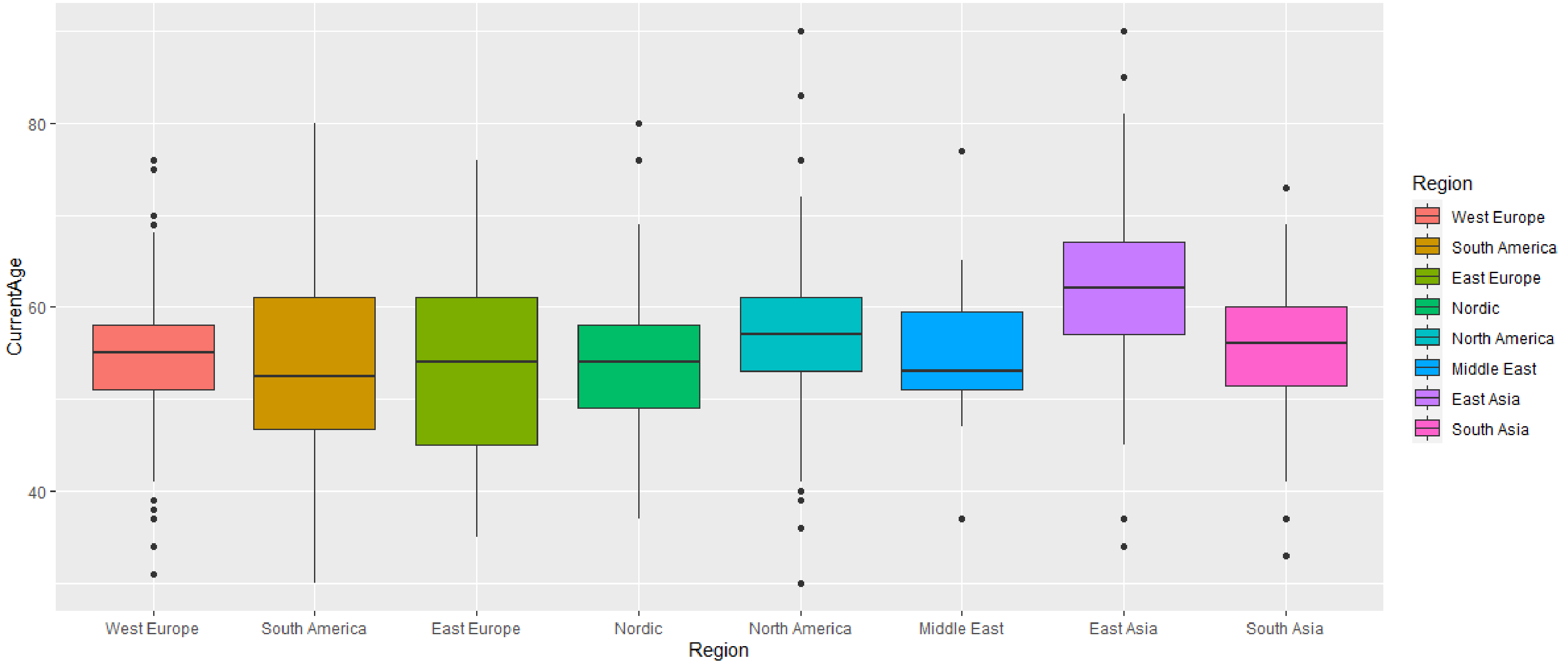

Note. Order is based on the main text version of this figure, for ease of comparison.

Figure S-2.

Business Leader Ages (Raw) by Culture Contrast. (Study 1).

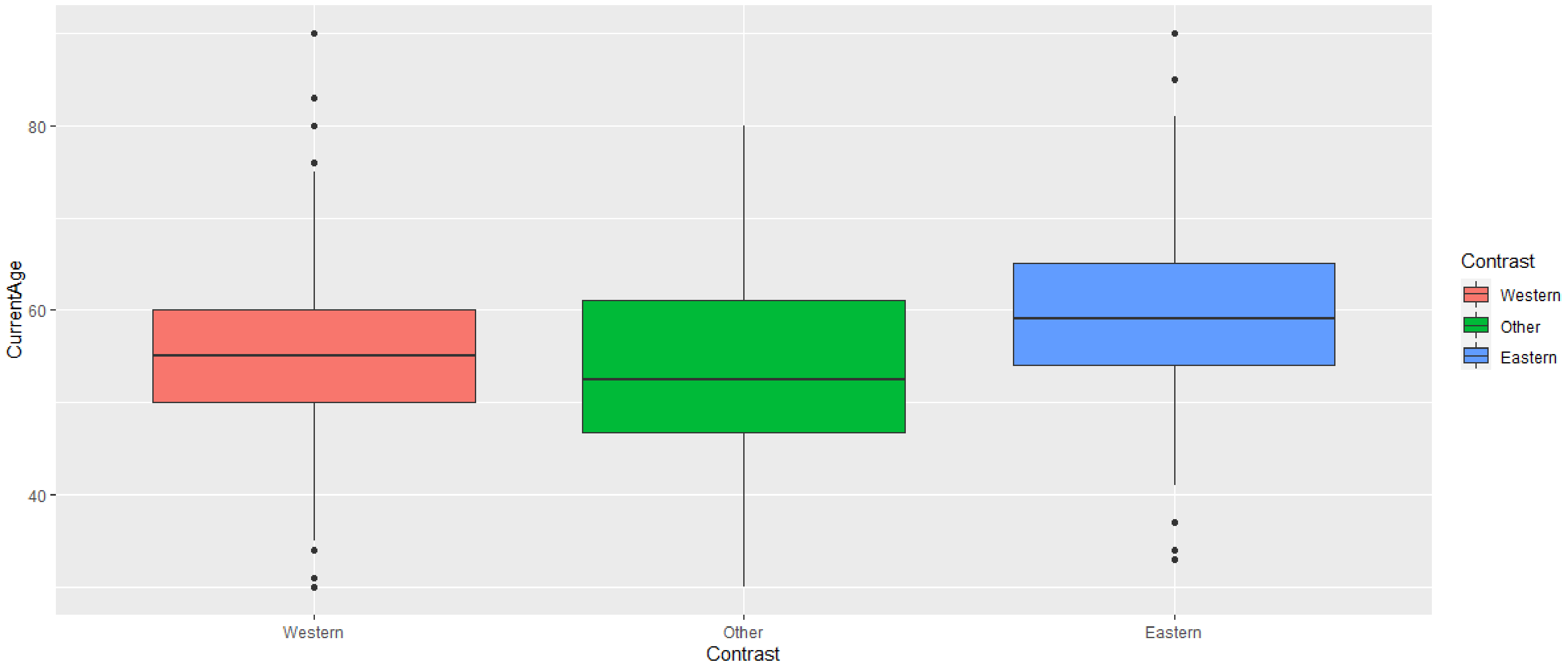

Figure S-3.

Political Leader Ages (Raw) by Geographical Region. (Study 2).

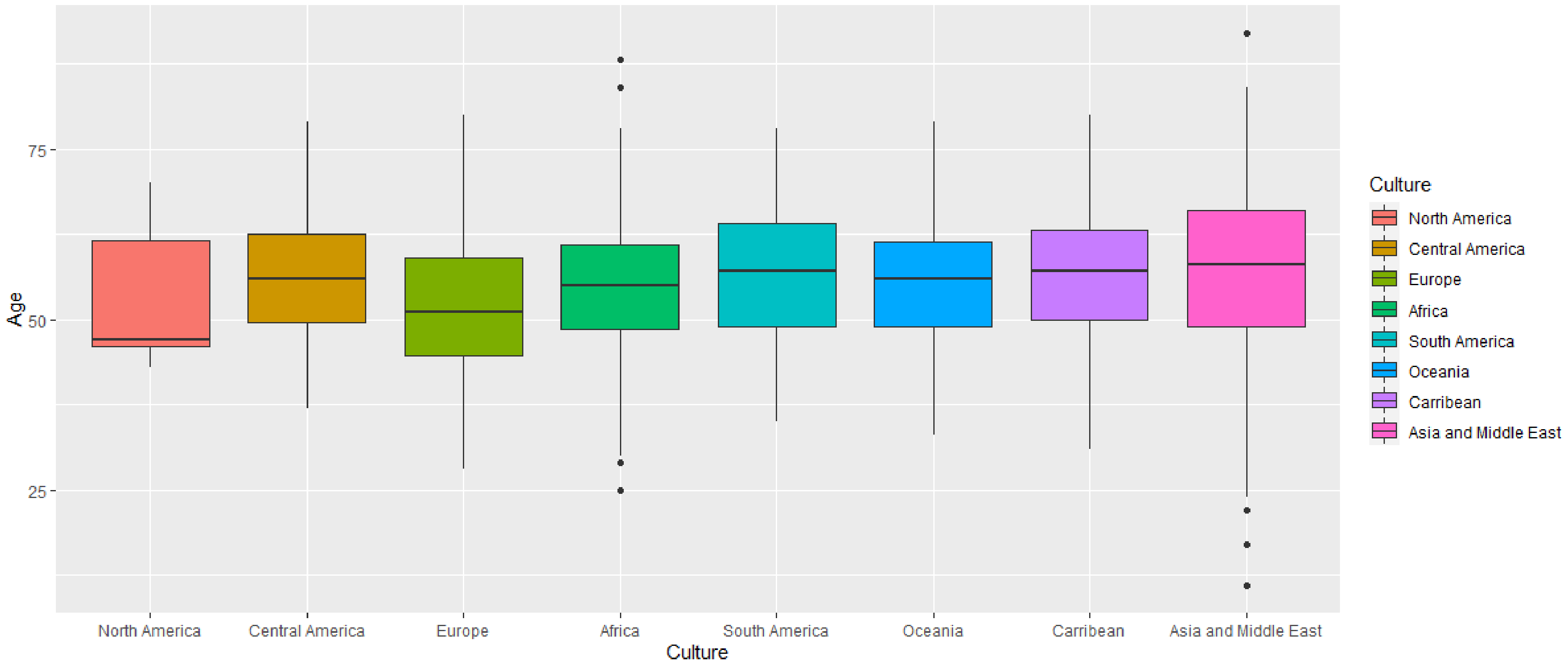

Figure S-4.

Political Leader Ages (Raw) by Culture Contrast. (Study 2).

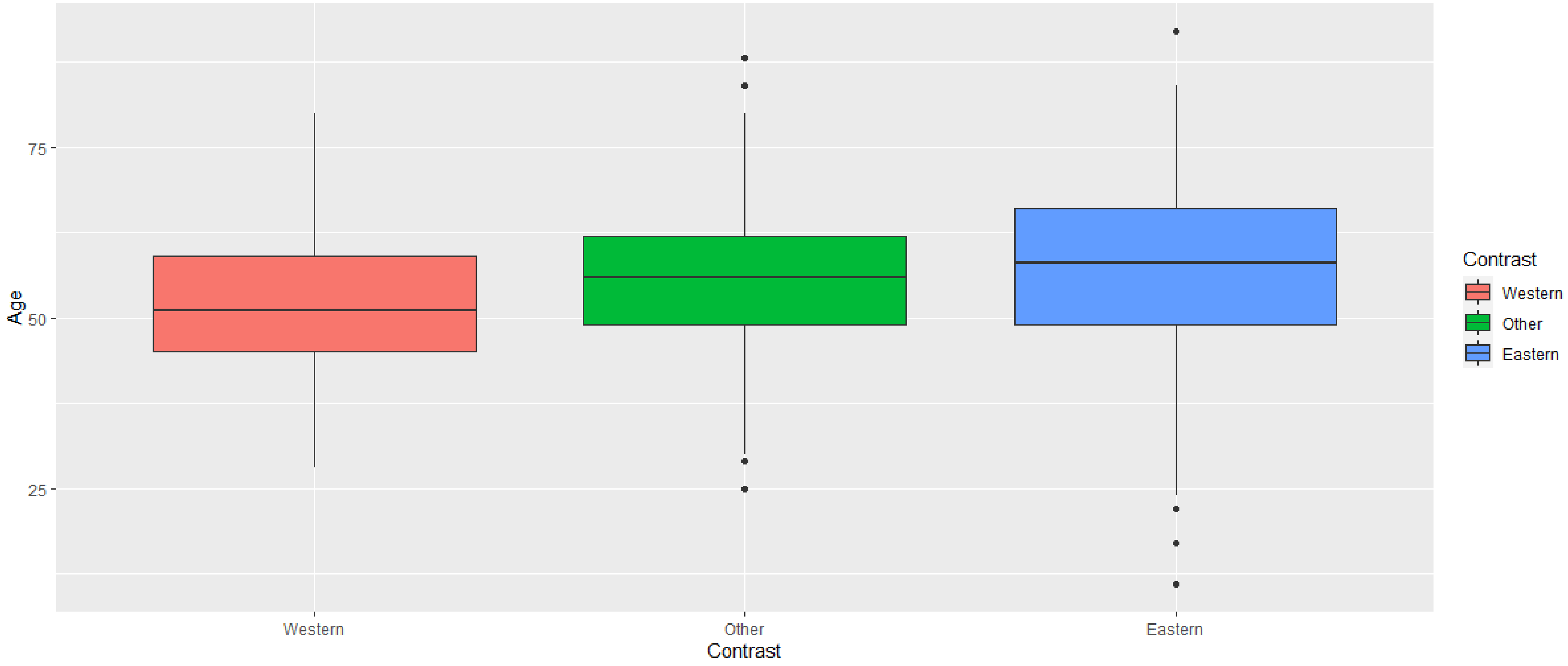

Supplement: Supplementary file 1 [file Data_Sheet_1.pdf]
